# A Case of Nephrectomy on Account of Nephrolithiasis and Pyonephrosis1Read before the Central New York Medical Association, Buffalo, N. Y., October 16, 1894.

**Published:** 1895-02

**Authors:** Herman Mynter

**Affiliations:** Professor of Surgery, Niagara University, Buffalo, N. Y.


### A CASE OF NEPHRECTOMY ON ACCOUNT OF NEPHROLITHIASIS AND PYONEPHROSIS.[1]

BY HERMAN MYNTER, M. D.,
Professor of Surgery, Niagara University, Buffalo, N. Y.

JOHN SETTER, 22 years of age, entered the Sisters' Hospital on August 24, 1894, with the following history:

He had complained for the last seventeen years of periodical attacks of pain in the back and left lumbar region. Up to five months ago the attacks occurred about once in six or eight weeks with free intervals, extending downwards in left scrotum and thigh and could only be relieved by hypodermics of morphia. They would suddenly cease and hours afterward he would pass, with great pain, a whitish, soft mass, which did not have the appearance of a stone, and then a profuse flow of scalding urine. During the last five months the above symptoms had increased in severity and become continuous. The patient had lost greatly in weight, suffered from profuse night sweats, loss of appetite, occasionally a dry cough. The parents were healthy, but one brother had died of consumption. At the examination the patient appeared extremely emaciated and cachectic, with protruding cheekbones and hectic flush. There was great tenderness from pressure in left lumbar region, but neither anteriorly nor posteriorly, standing or lying, could any swelling or enlargement of the kidney be discovered. He was also somewhat tender to pressure over the right lumbar region.

He passed about one quart of urine in the first twenty-four hours, half of which was pus The urine had a color like chocolate, specific gravity 1030, acid reaction and was loaded with albumin. Microscopically nothing was seen but pus corpuscles.

1. Read before the Central New York Medical Association, Buffalo, N. Y., October 16, 1894.

By cystoscopic examination under cocaine anesthesia and after preliminary irrigation of the bladder an excellent view was immediately obtained of the fundus of the bladder. The mucous membrane appeared perfectly normal. The orifice of the right ureter was seen as a small oblique slit through which a swirl of healthy urine passed every four or five seconds. Turning the cystoscope towards the orifice of the left ureter I saw what appeared to be a deep crater-formed ulcer, about two centimeters in diameter. Nothing appeared for two or three minutes, when suddenly a cohesive, thick, yellowish mass commenced to protrude through the crater, as thick apparently as a little finger. When about two inches of this mass had entered the bladder, it disappeared from view and was followed by a profuse discharge of yellowish pus, which quickly obscured the field. As it was evident from this examination that the patient suffered from a pyonephrosis of the left kidney, and that the right kidney was healthy, nephrectomy was advised, although his condition was so miserable that it seemed impossible he could survive such an operation. The operation was performed on August 8, 1894, under chloroform narcosis, the patient having been stimulated for a couple of days with hypodermics of strychnia, nitroglycerine, whisky and rectal alimentation. The pulse was then 130, weak; temperature had ranged between 101 and 103. The incision was made along the rectus spinal muscle, ending near crest of the ileum, combined with an oblique incision along the whole lower border of the twelfth rib. The kidney was almost wholly covered by the ribs, strongly attached to the colon and the surrounding tissue. During the process of enucleation, which was extremely difficult on account of the strong adhesions and its high position, my whole arm almost disappearing up to the elbow in the wound, a large abscess in the kidney itself was ruptured, from which a large amount of ill-smelling pus, estimated at one pint, escaped. The hilus was at last reached, the kidney brought out through the wound, two clamps attached on the hilus, in order to finish the operation quickly, and the kidney removed. The ureter was as large as a finger. The wound was disinfected with corrosive sublimate, loosely packed with iodoform gauze and partly sutured. The patient left the table in a state of profound shock, pulseless and with clammy perspiration, but by active stimulation he revived in a short time. The operation lasted about three-quarters of an hour.

The kidney preserved its general contour, but was enormously enlarged, its volume being about six times that of the normal. It measured seven inches in length, four in width and three in thickness. Its weight was twenty-eight ounces, but, as at least one pint of pus escaped, its weight must have been about forty-four ounces, equal to two and three-quarters pounds. The capsule was adherent and blended with the renal structure. The color in no place was that of a normal kidney, but that of fat and fibrous tissue.

The surface presented numerous large circumscribed swellings, which on palpation were soft and fluctuating, and on incision were found to contain a thick, greenish tenacious pus. On the posterior surface was a large abscess cavity, ruptured during the operation. On section, no normal kidney structure appeared. The upper third was a mass of multilocular abscesses, which intercommunicated and contained pus of the same character previously described. The middle third was a mass of fat and fibrous tissue. Lying in the latter was a large triangular-shaped stone, $1\frac{3}{4}$ x $\frac{3}{4}$ inches, of a greenish-brown color, irregular in shape and with rough surface. The lower third showed two abscesses, the larger being three-fourths of an inch in diameter. In one of the abscesses in the upper part a rough, irregular stone was found. The pelvis was greatly enlarged, full of pus and contained several cohesive, yellowish masses of necrotic tissue, impregnated with pus, like the one which was seen to pass with the cystoscope. Scarcely any traces of kidney tissue could be seen, and its function as a secreting gland had, probably, long since ceased. August 9th, temperature, 102 2-5 ; pulse, 130. He has passed 19 ounces of urine since the operation, the last of which is perfectly clear and normal. He is extremely weak and is given 1-60 strychnine and 1-100 nitroglycerine every three hours hypodermically. He has taken one quart of milk during the night.

August 10th, temperature, 101 ; pulse, 120 ; $28\frac{1}{2}$ ounces of clear, normal urine. The patient takes lots of nourishment and rests well.

August 11th, temperature, $99\frac{1}{2}$ ; pulse, 126 ; 31 ounces of urine. Clamps removed, and cavity irrigated and loosely packed with iodoform gauze.

August 12th, temperature, 99 ; pulse, 108 ; 27 ounces of urine. Patient takes solid food.

August 13th, temperature, 100 ; pulse, 110 ; 32 ounces of urine.

He gradually improved, passed from 35 to 45 ounces of urine daily ; pulse and temperature became normal. On August 23d, the dressing was found saturated with fecal matter, a fistula evidently having formed into the colon, but it healed in a couple of weeks.

On August 25th the patient was sitting up; the large cavity had contracted so that only a small granulating fistula was left. His weight had increased to 105 pounds; he felt perfectly well and was discharged to his home September 22d. Weight, 137 pounds, a gain of 36 pounds. Patient a picture of perfect robust health; urine normal; fistula closed.

October 1st, weight, 140 pounds. October 12th, weight, 147 pounds. The patient has gone to work in a machine shop.

The interest in this case is the ease with which the diagnosis was made by the aid of the electrical cystoscope, and particularly the demonstration that the right kidney was healthy and, therefore, an operation advisable. Only by operative means in two other ways —an explorative laparatomy or an explorative suprapubic cystotomy with catheterizing of ureters—could the diagnosis be cleared up, and there is no comparison between these methods and the simple cystoscopic examination, done without any danger to the exhausted patient. Cystoscopy necessitated, of course, that the bladder shall be able to contain five ounces of clear fluid, and that the cystoscope shall be able to enter the bladder. Luckily both indications were fulfilled in this case.

A second point of interest was the impossibility of feeling and palpating the enormous kidney before operation. I can only explain this by the strong adhesions of the lower segment of the kidney to the colon and surrounding tissue, by which the kidney was forced to enlarge upward and forward below the diaphragm. A third point of interest was the long time—seventeen years—in which the patient had suffered from nephrolithiasis without a distinct diagnosis having been made, and the rapid and perfect recovery that followed the removal of the diseased organ, the patient in seven weeks gaining forty-five pounds in weight. I have during the last three years performed nephrectomy four times. Two operations were done on account of tuberculous kidneys. One of these died on the fourth day of suppression of urine, and the post-mortem examination showed the remaining kidney to be tuberculous. A cystoscopic examination would in this case probably have shown that the operation was contra-indicated. The second case recovered, but died half a year later of general tuberculosis of the urinary organs. The third operation was performed in October, 1892, on account of a large sarcoma of the left kidney in a lady fifty years of age. The patient recovered, and so far, two years after the operation, no relapse has occurred and the patient is in perfect health.

